# Comparative clinical impact of low-curvature and normal-curvature titanium mesh in cranioplasty: a retrospective analysis of patient outcomes

**DOI:** 10.3389/fsurg.2025.1438307

**Published:** 2025-02-07

**Authors:** Shengkai Yang, Weihua Chen, Hongwei Teng, Lei Zhang, Kangkang Ji, Hai Zhou

**Affiliations:** ^1^ Department of Neurosurgery, Binhai People’s Hospital Affiliated to Kangda College, Nanjing Medical University, Yancheng, China; ^2^ Department of Central Laboratory, Binhai People’s Hospital Affiliated to Kangda College, Nanjing Medical University, Yancheng, China

**Keywords:** hypertensive intracerebral hemorrhages, low-curvature titanium mesh, normal-curvature titanium mesh, cranioplasty, PSM

## Abstract

**Objective:**

This study aimed to evaluate the clinical utility of two types of cranioplasty surgery involving low-curvature and normal-curvature titanium mesh, respectively.

**Methods:**

The clinical data were retrospectively collected from patients undergoing skull defect repair surgery between January 2021 and December 2022. The clinical outcomes associated with the two surgical approaches were compared and analyzed.

**Results:**

A total of 67 patients who underwent skull defect repair surgery were enrolled, with 22 in the low-curvature titanium mesh group and 45 in the normal-curvature titanium mesh group. Both before and after propensity score matching (PSM) analysis, the hospital stay for the low-curvature titanium mesh group was significantly shorter than that for the normal-curvature mesh group (Before: 9.14 ± 2.64 vs. 12.51 ± 4.15, *P* = 0.001; After: 9.44 ± 2.83 vs. 12.13 ± 4.40, *P* = 0.048). The low-curvature group exhibited lower overall hospitalization costs than the normal-curvature group (Before: 23500. ± 900. vs. 24,900. ± 1,100., *P* < 0.001; After: 23,300. ± 800. vs. 24,100. ± 1,000., *P* = 0.026). Moreover, satisfaction with molding (Before: 4.23 ± 0.75 vs. 3.18 ± 0.81, *P* = 0.001; After: 4.13 ± 0.72 vs. 3.25 ± 0.78, *P* < 0.001), Karnofsky's Performance Status score (Before: 93.32 ± 1.67 vs. 90.38 ± 3.50, *P* = 0.001; After: 93.56 ± 1.75 vs. 91.00 ± 3.78, *P* < 0.001), and Quality of Life score (Before: 52.95 ± 2.13 vs. 50.18 ± 3.54, *P* = 0.001; After: 53.31 ± 2.12 vs. 50.38 ± 4.23, *P* = 0.001) were significantly higher in the low-curvature titanium mesh group than the normal-curvature titanium mesh group.

**Conclusions:**

Applying low-curvature titanium mesh for skull repair effectively shortens the hospital stay, reduces overall hospitalization costs,enhances patient satisfaction with surgical modeling, and improves the postoperative functional status and quality of life of patients undergoing neurosurgery. These advantages warrant further clinical promotion.

## Introduction

Severe craniocerebral injuries and hypertensive intracerebral hemorrhages are associated with higher mortality and disability rates (1). Traumatic brain injuries lead to 1.5 million hospitalizations and 57,000 deaths in Europe annually. Hypertensive intracerebral hemorrhages exhibit a sudden onset, rapid progression, and a high fatality rate of 35%–52% within 30 days of onset, imposing a substantial burden on patients, their families, and society (2). Decompressive craniotomy, commonly performed on the frontotemporal roof, represents a primary method to alleviate refractory malignant cerebral edema and intracranial hypertension resulting from severe craniocerebral trauma and hypertensive intracerebral hemorrhage (3–6). When the patients attain a generally stable condition, and intracranial edema has largely subsided, the imperative for cranioplasty (CP) arises to reconstruct skull defects and enhance neurological function (7, 8).

CP employs various materials, including titanium skull repair materials, autologous bone, polymethyl methacrylate synthetic materials, and bioactive materials such as hydroxyapatite. Given its high strength, lightweight nature, biocompatibility, corrosion resistance, and malleability, titanium alloy for skull repair enjoys widespread use in clinical practice (9). Recent studies indicate that CP restores the original appearance of patients and alleviates the psychological burden associated with skull defects. Moreover, it enhances the cerebral blood flow and promotes nerve function recovery. Early CP may yield more favorable outcomes (10–12). Previous investigations have highlighted that decompressive craniotomy frequently correlates with abnormal glucose metabolism, compromised cerebral blood flow, and alterations in cerebrospinal fluid circulation (13, 14). Recent reports underscore the occurrence of severe complications, such as challenging incision healing, incision infection, epilepsy, facial nerve injury, exposure to skull repair materials, temporal muscle atrophy, and the need for multiple remedial operations; thereby, some patients have to remove titanium alloy material.

CP-related complications are frequently attributed to blood supply, tension, and dead space beneath the patch material of the scalp. A low-central-point titanium alloy plate and mesh were utilized to mitigate scalp tension, improve the blood supply of the skin at the defect site, and minimize dead space under the scalp. This design aims to bring the titanium alloy plate close to the dural membrane and temporal muscle below. Subsequently, we conducted a retrospective analysis of clinical data from patients with skull defects treated using low-curvature and normal-curvature titanium mesh, comparing the clinical benefits and prognoses of the two surgical modalities.

## Materials and methods

### Patients

A retrospective analysis was conducted on 86 adult patients with skull defects who underwent skull repair surgery due to cerebral hemorrhage and craniocerebral trauma in the Department of Neurosurgery from Binhai County People's Hospital between January 2021 and December 2022. The inclusion criteria comprised patients identified with cerebral hemorrhage or craniocerebral trauma through imaging examination, aged 20 years or older, and willing to undergo surgery. Exclusion criteria included incomplete clinical data, missing information in patient follow-ups, and severe medical conditions potentially affecting the length of hospital stay.

### Surgical methods of cranioplasty

**Figure 1:**
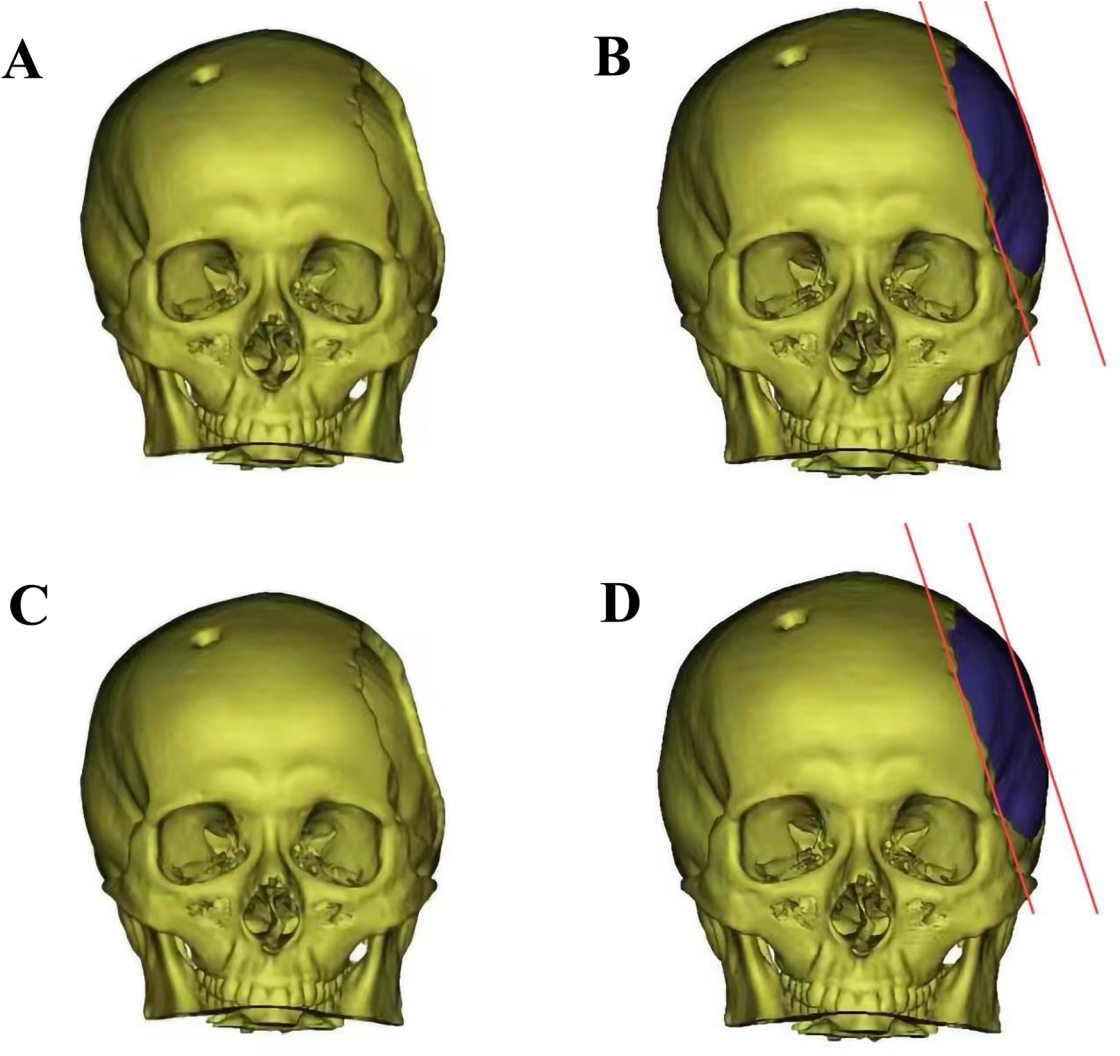
The diagram of skull before and after cranioplasty. **(A)** Left temporal skull defect before titanium mesh cranioplasty; **(B)** Three-dimensional model of low-curvature titanium mesh designed by factory; **(C)** Left temporal skull defect before titanium mesh cranioplasty; **(D)** Three-dimensional model of normal-curvature titanium mesh.

The control group received normal-curvature titanium mesh for skull repair. In contrast, the observation group underwent skull repair with titanium mesh and a titanium alloy plate featuring a 5 mm central point compression of normal curvature (Figure 1). All patients underwent preoperative double-source thin-slice head computerized tomographic (CT) scans and three-dimensional reconstructions. Skull defect data were obtained and sent to the manufacturing company [Kontour (Xi'an) Medical Technology Co., Ltd], which customized titanium mesh of varying curvatures by comparing images of the healthy and defective sides. The titanium mesh was composed of a bone plate and a bone screw. The bone plates were made of TA2 pure titanium material in accordance with Chinese GB/T 13810 standard, and the bone screws were made of TC4 titanium alloy material in accordance with Chinese GB/T 13810 standard. Pure titanium and titanium alloy products have no color on the surface and are packaged with sterilization. As a preventive measure against infection, antibiotics were routinely administered 30 min before the surgical procedure. Under successful general anesthesia, the patient's head was positioned on the healthy side in the supine position. The skull repair material was initially immersed in vancomycin water, and the dura was carefully preserved intraoperatively. Any damage was closely sutured to prevent postoperative cerebrospinal fluid leakage. The titanium mesh was then covered and secured with titanium nails. Subcutaneous drainage was maintained for 3 days postoperatively, with removal based on drainage flow criteria (daily drainage volume <50 ml). Stitches were removed 7–10 days after surgery.

### Study variables

The medical records of the enrolled patients were retrospectively analyzed. The demographic information (sex and age), preoperative clinical data (etiology of bone flap removal, defect area, skull defect site, skull defect time), operative data (operation time, intraoperative blood loss, difficulty evaluation), postoperative nursing conditions and aesthetics, and postoperative complications were collected. Hospital stay duration for both groups was recorded, and patient satisfaction after CP was assessed through telephone follow-up, categorized as satisfaction, relative satisfaction, general satisfaction, and dissatisfaction. Based on patient journals, demographic characteristics, including age (<50 years old, ≥50 years old) and body mass index (BMI) (<24 kg/m^2^, ≥24 kg/m^2^), were considered.

Karnofsky's Performance Status (KPS) and quality-of-life (QOL) scores were evaluated. KPS score is widely utilized in patients with cancer, determines their functional status and predicts the likelihood of adverse postoperative outcomes or risks. Ranging from 0 to 100, a score of 0 signifies the patient's demise, 80 to 89 indicates the presence of disease symptoms with the ability to perform everyday activities independently, 90 to 99 suggests mild signs or symptoms of the disease with the capability to perform regular activities, and 100 signifies normal health. A higher score correlates with better physical condition and increased tolerance to adverse reactions post-treatment (15). QOL score is commonly employed in patients with cancer, and it is a crucial tool for evaluating the quality of life and overall health status. The QOL evaluation index encompasses 12 aspects, each categorized into five levels based on the quality of life. The total QOL score is 60 points, with less than 20 indicating poor quality of life, 21–30 as poor quality, 31–40 as average quality, 41–50 as better quality, and 51–60 as good quality of life (16).

### Statistical analysis

SPSS version 27.0 and GraphPad Prism 8.3.0 statistical software were employed for analysis. Measurement data were compared using a t-test, and counting data were compared using Pearson *χ*^2^ test, continuity correction *χ*^2^ test, or Fisher's Exact test based on data characteristics. Statistical significance was set at *P* < 0.05. The 1:1 propensity score matching (PSM) was implemented to minimize inter-group variable selection bias. PSM analysis was used to adjust for differences between patients with low-curvature and normal-curvature titanium mesh, adjusting for demographic information (sex and age), preoperative clinical data (etiology of bone flap removal, defect area, skull defect site, skull defect time), operative data (operation time, intraoperative blood loss, difficulty evaluation), postoperative nursing conditions and aesthetics, and postoperative complications.

## Results

### Demographic and clinical characteristics of patients

**Figure 2:**
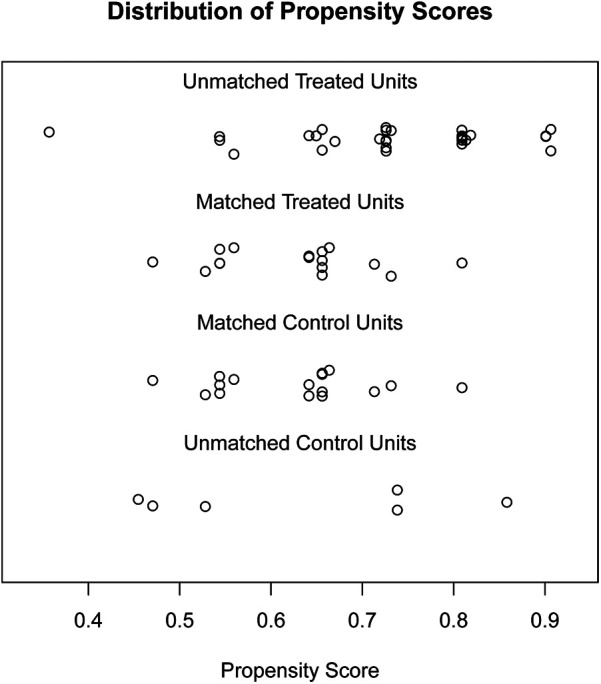
PSM analysis depicting the standardized mean difference results for various variables. PSM, propensity score matching.

This study identified 86 eligible patients with skull defects between January 2021 and December 2022, with 22 in the low-curvature titanium mesh group and 45 in the normal-curvature group. Table 1 displayed the distribution, showing 16 men and six women in the low-curvature group, and 28 men and 17 women in the normal-curvature group. Fifteen patients in the low-curvature group were significantly older than 50 years, and seven were <50 years; 19 in the normal-curvature group were considerably older than 50 years, and 26 were <50 years (*P* = 0.046). Sixteen patients in the low-curvature group had a BMI >24 and six had ≤24; 20 in the normal-curvature group had a BMI between 24 and 25, with significantly different results (*P* = 0.030). In the low-curvature group, two complications occurred, compared to seven in the normal-curvature group, demonstrating no significant difference (*P* = 0.728). Furthermore, there were no significant differences between the two groups regarding the cause, site, and area of skull defects, repair operation time, intraoperative blood loss, surgical and care difficulty, and aesthetics. The 1:1 PSM method (Figure 2, Table 2) was employed to ensure baseline variable balance, enrolling 32 patients in the final study, with 16 cases each in the low-curvature and normal-curvature titanium mesh group. No significant differences were observed between the two groups in age, sex, BMI, cause of skull defect, skull defect site, skull defect area, skull repair operation time, intraoperative blood loss, surgical and care difficulty, aesthetics, and occurrence of complications.

**Table 1:** Baseline demographic and clinical characteristics of cranioplasty in the low-curvature and normal-curvature groups before PSM.

Characteristics	Total *n* = 67	Low curvature *n* = 22	Normal curvature *n* = 45	*P* value
Age, *n* (%)				0.046
<50	33 (49.3%)	7 (31.8%)	26 (57.8%)	
≥50	34 (50.7%)	15 (68.2%)	19 (42.2%)	
Sex, *n* (%)				0.400
Female	23 (34.3%)	6 (27.3%)	17 (37.8%)	
Male	44 (65.7%)	16 (72.7%)	28 (62.2%)	
BMI, *n* (%)				0.030
≤24	31 (46.3%)	6 (27.3%)	25 (53.2%)	
>24	36 (53.7%)	16 (72.7%)	20 (46.8%)	
Cause of defects, *n* (%)				0.536
Craniocerebral injury	46 (68.7%)	14 (63.6%)	32 (71.1%)	
Cerebral hemorrhage	21 (31.3%)	8 (36.4%)	13 (28.9%)	
Defect site, *n* (%)				0.548
Left	30 (44.8%)	11 (50.0%)	19 (42.2%)	
Right	37 (55.2%)	11 (50.0%)	26 (57.8%)	
Difficulty of surgery, *n* (%)				0.290
Easy	14 (20.9%)	7 (31.8%)	7 (15.6%)	
Normal	37 (55.2%)	11 (50.0%)	26 (57.8%)	
Hard	16 (23.9%)	4 (18.2%)	12 (26.6%)	
Defect area, mm^2^	91.07 ± 27.26	91.64 ± 27.12	90.71 ± 27.62	0.897
Defect time, days	5.64 ± 2.92	5.591 ± 1.40	5.667 ± 3.44	0.154
Surgery time, min	129.20 ± 29.59	127.00 ± 28.88	130.20 ± 30.20	0.758
Intraoperative bleeding, ml	172.40 ± 79.16	175.90 ± 70.35	170.70 ± 83.84	0.490
Difficulty of care, *n* (%)				0.043
Easy	32 (47.8%)	15 (68.2%)	17 (37.8%)	
Normal	31 (46.3%)	7 (31.8%)	24 (53.3%)	
Hard	4 (5.9%)	0 (0%)	4 (8.9%)	
Aesthetics, *n* (%)				0.225
Good	27 (40.3%)	12 (54.5%)	15 (33.3%)	
Normal	34 (50.7%)	9 (40.9%)	25 (55.6%)	
Poor	6 (9.0%)	1 (4.6%)	5 (11.1%)	
Complication, *n* (%)				0.728
No	58 (86.6%)	20 (90.9%)	38 (84.4%)	
Yes	9 (13.4%)	2 (9.1%)	7 (15.6%)	
Duration of hospitalization, days	11.40 ± 4.03	9.14 ± 2.64	12.51 ± 4.15	<0.001
Titanium mesh cost, ¥	15,800. ± 900.	16,000. ± 900.	15,900. ± 1,000.	0.658
Overall hospitalization costs, ¥	24,100. ± 1,000.	23,500. ± 900.	24,900. ± 1,100.	<0.001
Satisfaction with molding, *n* (%)				<0.001
Satisfactory	12 (17.9%)	9 (40.9%)	3 (6.7%)	
Relatively satisfactory	19 (28.4%)	9 (40.9%)	10 (22.2%)	
Generally satisfactory	28 (41.8%)	4 (18.2%)	24 (53.3%)	
Less satisfactory	8 (11.9%)	0 (0%)	8 (17.8%)	
KPS score	91.34 ± 3.32	93.32 ± 1.67	90.38 ± 3.50	<0.001
QOL score	51.09 ± 3.39	52.95 ± 2.13	50.18 ± 3.54	<0.001

Median and interquartile range (IQR) and *n* (%) were reported for continuous and categorical variables, respectively. BMI, Body Mass Index; KPS, Karnofsky's Performance Status; QOL, quality-of-life.

**Table 2:** Baseline demographic and clinical characteristics of cranioplasty in the low-curvature and normal-curvature groups after PSM.

Characteristics	Total (*n* = 32)	Low curvature (*n* = 16)	Normal curvature (*n* = 16)	*P* value
Age, *n* (%)				0.433
<50	9 (28.1%)	3 (18.7%)	6 (37.5%)	
≥50	23 (71.9%)	13 (81.3%)	10 (62.5%)	
Sex, *n* (%)				1.000
Female	5 (15.6%)	3 (18.8%)	2 (12.5%)	
Male	27 (84.4%)	13 (81.2%)	14 (87.5%)	
BMI, *n* (%)				0.473
>24	19 (59.4%)	11 (68.8%)	8 (50%)	
≤24	13 (40.6%)	5 (31.2%)	8 (50%)	
Cause of defects, *n* (%)				0.685
Craniocerebral injury	24 (75%)	11 (68.8%)	13 (81.2%)	
Cerebral hemorrhage	8 (25%)	5 (31.2%)	3 (18.8%)	
Defect site, *n* (%)				1.000
Left	13 (40.6%)	6 (37.5%)	7 (43.8%)	
Right	19 (59.4%)	10 (62.5%)	9 (56.2%)	
Difficulty of surgery, *n* (%)				0.611
Easy	8 (25%)	5 (31.3%)	3 (18.8%)	
Normal	14 (43.8%)	7 (43.8%)	7 (43.8%)	
Hard	10 (31.2%)	4 (24.9%)	6 (37.4%)	
Defect area, mm^2^	87.22 ± 24.47	88 ± 24.30	86.44 ± 25.41	0.860
Defect time, days	6.13 ± 3.03	5.63 ± 1.54	6.63 ± 4.05	0.969
Surgery time, min	129.20 ± 29.61	124.30 ± 29.38	134.10 ± 29.98	0.360
Intraoperative bleeding, ml	182.80 ± 76.55	175.60 ± 74.20	190.0 ± 80.58	0.719
Difficulty of care, *n* (%)				0.473
Easy	19 (59.4%)	11 (68.8%)	8 (50%)	
Normal	12 (37.5%)	5 (31.2%)	7 (43.8%)	
Hard	1 (3.1%)	0 (0%)	1 (6.2%)	
Aesthetics, *n* (%)				0.178
Good	14 (43.8%)	9 (56.3%)	5 (31.2%)	
Normal	16 (50%)	7 (43.7%)	9 (56.2%)	
Poor	2 (6.2%)	0 (0%)	2 (12.6%)	
Complication, *n* (%)				0.226
No	29 (90.6%)	16 (100%)	13 (81.3%)	
Yes	3 (9.4%)	0 (0%)	3 (18.7%)	
Duration of hospitalization, days	10.78 ± 3.88	9.44 ± 2.83	12.16 ± 4.40	0.049
Titanium mesh cost, ¥	15,700. ± 1,000.	16,000. ± 800.	15,800. ± 1,000.	0.761
Overall hospitalization costs, ¥	23,800. ± 900.	23,300. ± 800.	24,100. ± 1,000.	0.026
Satisfaction with molding, *n* (%)				0.032
Satisfactory	6 (18.8%)	5 (31.2%)	1 (6.2%)	
Relatively satisfactory	12 (37.6%)	8 (50%)	4 (25%)	
Generally satisfactory	12 (37.6%)	3 (18.8%)	9 (56.3%)	
Less satisfactory	2 (6.0%)	0 (0%)	2 (12.5%)	
KPS score	92.28 ± 3.17	93.56 ± 1.75	91.00 ± 3.77	0.022
QOL score	51.84 ± 3.61	53.31 ± 2.12	50.38 ± 4.22	0.019

Median and interquartile range (IQR) and *n* (%) were reported for continuous and categorical variables, respectively. BMI, Body Mass Index; KPS, Karnofsky's Performance Status; QOL, quality-of-life.

### Comparison of hospitalization duration, cost,and molding satisfaction

**Figure 3:**
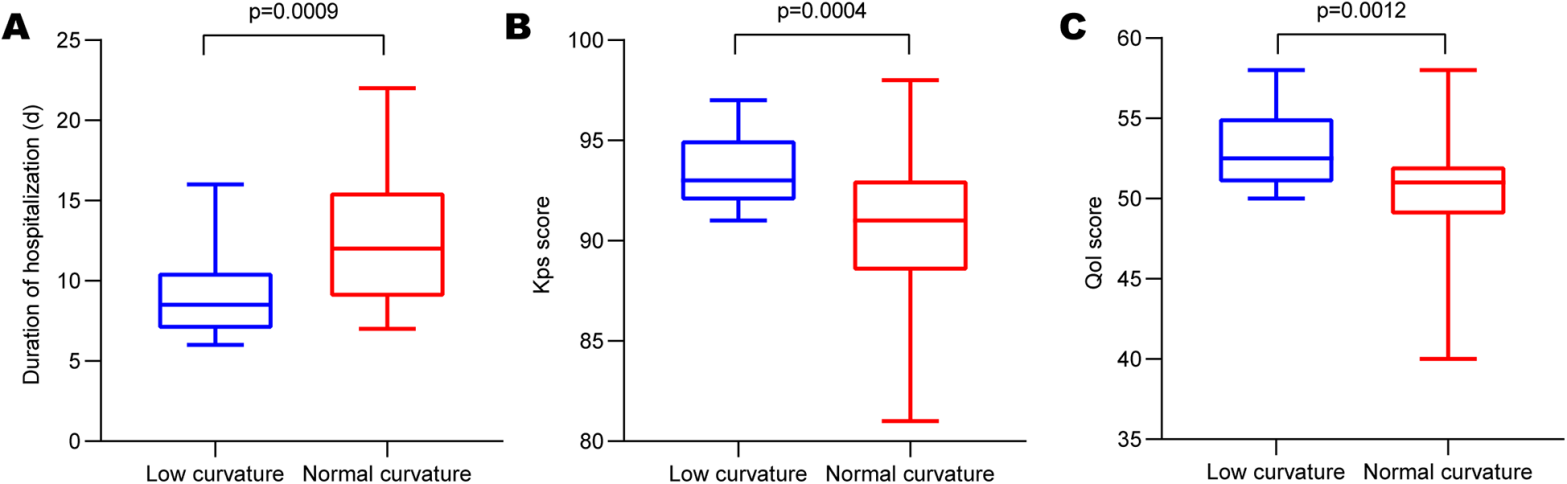
Comparison of hospitalization duration and molding satisfaction. **(A)** Differences in the expression of hospitalization duration; **(B)** and **(C)** KPS score and QOL score between the low-curvature and normal-curvature titanium mesh groups before PSM.

**Figure 4:**
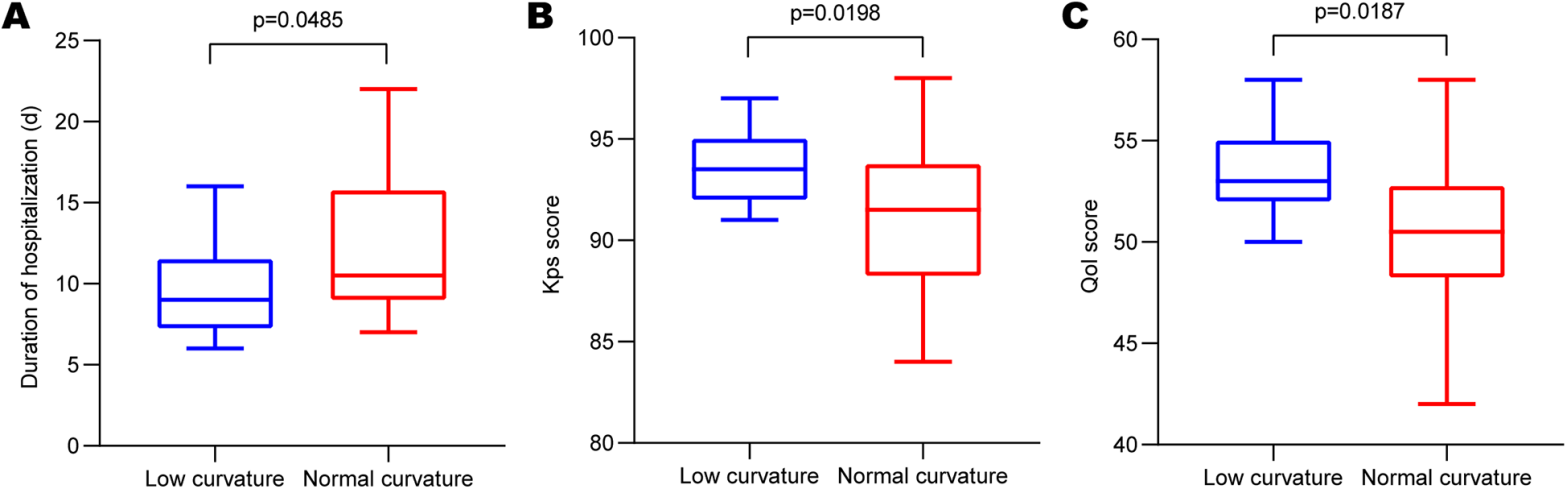
Comparison of hospitalization duration and molding satisfaction after PSM. **(A)** Differences in the expression of hospitalization duration after PSM; **(B)** and **(C)** KPS score and QOL score between the low-curvature and normal-curvature titanium mesh groups after PSM. PSM, propensity score matching; KPS, Karnofsky's Performance Status; QOL, quality-of-life.

We investigated plastic satisfaction and hospitalization duration for all patients with skull defects. As depicted in Figure 3A, a significant difference was observed between the two groups (low-curvature group: 9.136 ± 2.642 days, normal-curvature group: 12.51 ± 4.149 days, *P* < 0.001), with the low-curvature group exhibiting a shorter duration than the normal-curvature group. After PSM, differences persisted between the two groups (Figure 4A) (low-curvature group: 9.438 ± 2.830 days, normal-curvature group: 12.125 ± 4.400 days, *P* = 0.049), with the hospitalization time for the low-curvature group remaining lower than the normal-curvature group. Pre-PSM and after PSM, the low-curvature group exhibited lower overall hospitalization costs than the normal-curvature group (Pre-PSM: 23,500. ± 900. vs. 24,900. ± 1,100., *P* < 0.001; after PSM: 23,300. ± 800. vs. 24,100. ± 1,000., *P* = 0.026). Patient-satisfaction analysis (Table 1) revealed a significant difference between the two groups (low curvature group: nine cases reported being satisfied, nine were relatively satisfied, four were generally satisfied, and none were dissatisfied; normal-curvature group: three cases reported being satisfied, ten were relatively satisfied, 24 were generally satisfied, and eight were dissatisfied, *P* < 0.001), with the low-curvature group expressing greater patient satisfaction than the normal-curvature group. Differences persisted between the two groups after PSM (low-curvature group: five cases reported being satisfied, eight were relatively satisfied, three were generally satisfied, and none were dissatisfied; normal-curvature group: one case reported being satisfied, four were relatively satisfied, two were generally satisfied, and two were dissatisfied, *P* = 0.032), with the low-curvature group exhibiting higher patient satisfaction than the normal-curvature group.

### Comparison of KPS and QOL scores

Additionally, we performed KPS and QOL score assessments for all enrolled patients. Results revealed a significant difference in KPS scores between the two groups (Figure 3B) (low-curvature group: 93.32 ± 1.67, normal-curvature group: 90.38 ± 3.50, *P* < 0.001), with the KPS score higher in the low-curvature group than that of the normal-curvature group. Post-PSM, differences persisted between the two groups (Figure 4B) (low-curvature group: 93.56 ± 1.750, normal-curvature group: 91.00 ± 3.777, *P* = 0.022), and the KPS score of the low-curvature group remained superior to that of the normal-curvature group. There was a significant difference in QOL scores between the two groups (Figure 3C) (low-curvature group: 52.95 ± 1.67, normal-curvature group: 50.18 ± 3.537, *P* < 0.001), with the QOL score higher in the low-curvature group than that of the normal-curvature group. After PSM, differences persisted between the two groups (Figure 4C) (low-curvature group: 53.31 ± 2.12, normal-curvature group: 50.38 ± 4.225, *P* = 0.022), and the QOL score of the low-curvature group remained superior to that of the normal-curvature group.

## Discussion

In this retrospective analysis, the clinical benefits of the two surgical modalities were explored to determine the superiority of low-curvature titanium mesh CP. Pre-PSM, the low-curvature group exhibited significantly reduced hospital stays, overall hospitalization costs, superior satisfaction with plastic form, and higher KPS and QOL scores than the normal-curvature group. Similar outcomes persisted after 1:1 PSM for sex, age, BMI, defect cause, and defect site. These results showed that the significantly decreased hospital stay in the low-curvature group reduced hospital costs, alleviating the economic pressure and family burdens, and ultimately improved quality of life.

A number of synthetic materials have been used as alternatives to autologous bone flap, including metal (titanium), acrylic acid (polymethyl methacrylate), ceramic (calcium phosphate-based cement such as hydroxyapatite), and plastic (such as PEEK). The ideal cranioplasty material should promote osseointegration and be lightweight, aesthetically pleasing, durable, physiologically compatible, and cost effective (17). It has been suggested that autologous bone flap has irreplaceable advantages because the replacement of the original bone flap takes advantage of its natural biocompatibility and associated low risk of rejection, as well as the potential for reintegration with adjacent bone and subsequent growth with the patient. However, its translational application in bioengineering is still limited and aseptic bone flap resorption (BFR) is one of the most common long-term complications (18, 19). A meta-analysis investigated risk factors for resorption of aseptic bone flaps after cranioplasty with autologous bone flaps and showed that more bone flap fragments, traumatic brain injury, and younger age significantly increased the risk of resorption of aseptic bone flaps (20). In these patients, choosing a synthetic implant may be a reasonable option (19).

The widespread use of titanium alloy in skull repair surgery has been reported, and recent studies highlight the advantages of PEEK materials, such as high translucency, mechanical strength, good histocompatibility, and resistance to scalp heating in sunlight (21). PEEK materials have shown lower complications and implant failure rates in skull repair compared to titanium and autogenous bone. However, no significant difference was found between PEEK and titanium alloy in the incidence of complications post-CP and post-discharge (22), emphasizing the lack of evidence supporting the superiority of PEEK over titanium mesh repair, especially considering the substantially higher cost of PEEK materials. Overall, titanium alloy remains the preferred surgical material for skull repair procedures in patients.

While titanium alloy materials are widely utilized in neurosurgery, they present challenges, such as limited light transmission, high thermal conductivity, increased wear on covering tissues, and difficulties in intraoperative secondary molding (23). Postoperative complications, such as challenging incision healing, incision infections, epilepsy, facial nerve injuries, exposure of skull repair materials, and temporal muscle atrophy, manifest in some patients undergoing skull repair with titanium alloy materials, necessitating multiple remedial operations (24). In this study, there were 2 cases of complications in the low-curvature group, accounting for 9.1%. Both 2 patients had large skull defects and difficult healing issues. There were 7 cases of complications in the normal-curvature group, accounting for 15.6%, including 4 cases of postoperative infection and 3 cases of difficult healing issues. This study advocates using low-curvature titanium mesh to mitigate complications following skull repair. The reasons for this rationale lie in the low-curvature treatment of the titanium mesh used in skull repair, alleviating tension after scalp suturing to prevent issues like poor blood supply due to excessive tension and pulled scalp vessels, leading to difficult incision healing, scalp necrosis, and exposed titanium mesh. Moreover, the low curvature of the mesh minimizes the postoperative dead space under the scalp, lowering the incidence of postoperative epidural hematoma and intracranial infection, ultimately contributing to shortened postoperative recovery times, abridged hospital stays, and decreased overall hospitalization costs for patients.

This study reveals a shorter hospital stay for the low-curvature group than the normal-curvature group. Prior research has established a direct correlation between the length of hospital stay, postoperative complications, and the patients' baseline conditions (25). In the low-curvature group, improved surgical techniques reduce tension after scalp suturing, preventing serious complications such as poor scalp blood supply caused by traction, complicated incision healing, scalp necrosis, and titanium mesh exposure. Furthermore, the low curvature treatment decreases the space between the scalp and the dural membrane, minimizing postoperative dead space and reducing the likelihood of postoperative epidural hematoma and intracranial infection (26, 27). Consequently, this study affirms that the low-curvature titanium mesh repair group experiences a lower complication rate, leading to significantly shorter hospitalization, reduced hospitalization expenses, and improved neurological prognosis.

The KPS score is a predictive tool for adverse postoperative outcomes or risks, aiding medical personnel in evaluating treatment effectiveness and predicting patient survival. In this study, the KPS score of the low-curvature group surpasses that of the normal-curvature group. The reason behind the higher scores in the low-curvature group remains challenging to elucidate, particularly considering that both groups' scores post-skull repair exceed 80, indicating complete self-care for patients (28). In contrast, this study revealed that patients undergoing low-curvature titanium mesh repair experienced shorter hospital stays and fewer surgical complications than those with normal-curvature repair. This outcome is attributed to the low-curvature treatment of titanium mesh, which decreases scalp tension, improves scalp blood supply, and diminishes the space between the scalp and meninges. Low curvature titanium mesh was able to significantly improve KPS and whether it was associated with factors such as reduced postoperative pain and fewer complications.No other evidence supporting KPS as an influencing factor is found in existing literature, suggesting a potential avenue for exploration in subsequent studies.

The QOL score, commonly used in patients with tumor and post-neurosurgery evaluations (29), was higher in the low-curvature group than in the normal-curvature group. The results of this metric exhibited similarity between the two groups of patients undergoing surgery. However, the comprehensive nature of its scoring, encompassing 12 dimensions, including appetite, spirit, sleep, fatigue, pain, family and colleagues' understanding and cooperation, patients' perception of the disease, attitude toward treatment, daily life, treatment side effects, and facial expressions, rendered the results more robust and convincing than the classification of KPS scores. This study suggests that the primary reason for the difference between the two groups may be that postoperative pain in patients undergoing low-curvature titanium mesh skull repair is less than those with normal-curvature titanium mesh. The snugger fit resulting from the lower curvature of the skull repair reduces the tension of the incision skin, consequently alleviating patient pain. This pain reduction is accompanied by increased appetite, improved sleep quality, and a more positive attitude towards subsequent treatment. Also, patient's psychological state and postoperative rehabilitation environment may influence the differences in scores. The surgical procedures explored in this study are poised to receive further validation through expanded case studies.

To our knowledge, this is the first study to compare the clinical benefits and prognosis of low-curvature titanium mesh and normal-curvature titanium mesh in the treatment of cranioplasty. However, this study has several limitations inherent to its retrospective design. Primarily, it is a single-center, small-sample investigation, which limits the generalizability of the findings and the applications in other settings. We will needa multi-center, prospective, large-sample study for comprehensive validation in the future. The retrospective analysis employed may introduce selection bias. Additionally, the limited sample size may limit the statistical power to detect differences, particularly in less common complications or rare events. Furthermore,the study does not provide the dynamic post-discharge follow-up, a detailed examination of postoperative complications, such as infection, seizures, epidural hematoma, hardware failure, and subdural effusion, was not conducted.

## Conclusion

This retrospective cohort study highlights that skull repair utilizing low-curvature titanium mesh shortens hospital stay effectively, improves patient satisfaction with surgical outcomes, and improves the postoperative functional status and quality of life for neurosurgically treated patients. These promising findings warrant further clinical exploration and promotion.

## Data Availability

The original contributions presented in the study are included in the article/Supplementary Material, further inquiries can be directed to the corresponding author.
